# PanGFR-HM: A Dynamic Web Resource for Pan-Genomic and Functional Profiling of Human Microbiome With Comparative Features

**DOI:** 10.3389/fmicb.2018.02322

**Published:** 2018-10-08

**Authors:** Narendrakumar M. Chaudhari, Anupam Gautam, Vinod Kumar Gupta, Gagneet Kaur, Chitra Dutta, Sandip Paul

**Affiliations:** ^1^Structural Biology and Bioinformatics Division, CSIR-Indian Institute of Chemical Biology, Kolkata, India; ^2^Department of Pharmacoinformatics, National Institute of Pharmaceutical Education and Research, Kolkata, India

**Keywords:** comparative genomics, database, functional profile, human microbiome, pan genome, web resource

## Abstract

The conglomerate of microorganisms inhabiting various body-sites of human, known as the human microbiome, is one of the key determinants of human health and disease. Comprehensive pan-genomic and functional analysis approach for human microbiome components can enrich our understanding about impact of microbiome on human health. By utilizing this approach we developed PanGFR-HM (http://www.bioinfo.iicb.res.in/pangfr-hm/) – a novel dynamic web-resource that integrates genomic and functional characteristics of 1293 complete microbial genomes available from Human Microbiome Project. The resource allows users to explore genomic/functional diversity and genome-based phylogenetic relationships between human associated microbial genomes, not provided by any other resource. The key features implemented here include pan-genome and functional analysis of organisms based on taxonomy or body-site, and comparative analysis between groups of organisms. The first feature can also identify probable gene-loss events and significantly over/under represented KEGG/COG categories within pan-genome. The unique second feature can perform comparative genomic, functional and pathways analysis between 4 groups of microbes. The dynamic nature of this resource enables users to define parameters for orthologous clustering and to select any set of organisms for analysis. As an application for comparative feature of PanGFR-HM, we performed a comparative analysis with 67 *Lactobacillus* genomes isolated from human gut, oral cavity and urogenital tract, and therefore characterized the body-site specific genes, enzymes and pathways. Altogether, PanGFR-HM, being unique in its content and functionality, is expected to provide a platform for microbiome-based comparative functional and evolutionary genomics.

## Introduction

The variety of microorganisms inhabiting different body-sites of human – is one of the key determinants of human health and disease. Recent emergence of metagenomic approaches, empowered by the technical and conceptual advancements in low-cost, high-throughput sequencing methodologies have enabled the scientific community to understand the genetic/functional diversity of the “healthy microbiome” components, a crucial step for identifying the microbial species that are implicated in disease (Reid et al., 2011; Gupta et al., 2017). The vast resource of microbial reference genomes from different body-sites of healthy humans, available at Data Analysis and Coordination Center of Human Microbiome Project (HMP-DACC)^1^, provides the scientific community an opportunity to comprehend the genomic landscape and thus functional potential of any particular group of organisms in various body habitats (NIH HMP Working Group et al., 2009; Human Microbiome Project Consortium, 2012).

One of the major bioinformatic frameworks that have been proven to be useful and informative in comparative analysis of multiple microbial genomes is the ‘pan-genome’ approach developed by Tettelin et al. (2005). Pan-genome of a given species/taxon represents the complete set of non-redundant genes from its representative genomes and is comprised of three parts: core genes (representatives from all genomes), accessory genes (representatives from two or more genomes, not all) and genome specific genes. The pan-genomic profiling and subsequent systemic functional annotation at various taxonomic levels, varying from within-species community to cross-species communities at intra-/inter-habitat level, offer evolutionary insights and potential functional importance of any group of microorganisms. Moreover, various reports reveal that the comparative pan-genome analysis has tremendous potential for offering new perspective on the species diversity and adaptive strategies of human microbiome in body-site specific manner (Rasko et al., 2008; Conlan et al., 2012; Gupta et al., 2015; Bakshi et al., 2016; Duranti et al., 2016). Therefore, a comprehensive resource of human microbiome providing in-depth pan-genomic analysis of strains at various taxonomic levels, with subsequent estimation of the functional repertoire from same or different body-sites along with comparative analysis approach will be of great interest.

The existing database tools for pan-genome analysis of microbes like, MetaRef (Huang et al., 2014), MicroScope platform (Vallenet et al., 2017), and EDGAR 2.0 (Blom et al., 2016) provide basic pan-genomic information about the microbes in general but lack features like user-defined selection of strains or isolation body-site from human, in-depth strain wise pan-genomic details of shared genes, and strain specific presence/absence of genes along with their functional profiling. Also, there is no such resource which allows users to investigate/compare pan-genomes of multiple user defined groups within human microbiome strains. To this end, we developed PanGFR-HM – Pan-Genomic and Functional Repertoire of Human Microbiome components – an online dynamic resource that systematically integrates the functional and compositional characteristics of complete gene repertoire of 1293 reference bacterial and archaeal genomes from HMP-DACC. It offers options for pan-genomic analysis, potential functional analysis using Clusters of Orthologous Genes (COG) and Kyoto Encyclopedia of Genes and Genomes (KEGG), and comparative analyses for any possible combinations of genomes. The features for pan-genome analysis provide information about core, accessory and unique gene families among a user defined set of genomes, which can belong to a specific taxonomical clade or body-site. PanGFR-HM allows the users to explore the genomic and functional diversity, potential lateral gene transfer events and phylogenetic relationships between human associated microbial genomes, which are not provided by any existing public domain computational resources. Exceptionally, within a user defined set of genomes, this resource provides information about probable gene loss events, i.e., the genes exclusively absent from a specific genome but present in all other genomes. Also, significant over/under representation of KEGG/COG functional categories in different gene families (core, accessory, unique) are provided for that dataset. Most importantly, this resource enables users to perform comparative analysis between different groups of microbes (based on taxonomy and/or body-site) for common as well as group specific functional and gene-family architectures. All the results can be accessed freely through an online web-interface, interactively and can be downloaded for further analysis. We envision that, PanGFR-HM, being unique in its content and functionality, will greatly facilitate the progress of microbiome-based evolutionary research, clinical application of microbial genomics and create footprints for future studies on the composition-activity relationship of the human microbiome components.

## Materials and Methods

### Overview of PanGFR-HM

PanGFR-HM serves as an ample and appropriate resource for exploring the genomic and functional repertoire and diversity, phylogenetic relationships among human associated microbial genomes by providing numerous attributes not available in any existing computational resources. All 1293 strains belong to 8 major bacterial/archaeal phyla, i.e., *Actinobacteria, Bacteroidetes, Firmicutes, Fusobacteria, Proteobacteria, Spirochaetes, Synergistetes*, and *Euryarchaeota*. At genus level, these genomes represent 187 different defined genera (see **Supplementary Table S1**). These microbes, as part of human microbiome, comprise mostly of bacteria derived from distinct body-sites of human (Detailed list of microbial species is provided in **Supplementary Table S1**). Gene families (gene clusters) generated from all annotated proteins from complete genomes of these microbes were integrated into a database, where pan-genomic details of any subset of these microbial strains belonging to specific taxonomical clade or body-site, can be dynamically retrieved.

PanGFR-HM provides the pan-genomic profile for genomes of the interest based on user defined sequence identity criteria for protein sequences (ranging from 40 to 90%) for detection of orthologous clusters. The pan-genome profile comprises of comprehensive information about core gene families, accessory gene families, gene families with genome wise exclusive presence and absence, and prediction of nature of pan-genome (open/close) with statistics. PanGFR-HM integrates additional features for reconstructing the phylogenetic relationships among selected genomes based on concatenated core genes (users can select 10, 20, 30, 50, 70, or 100 random core genes for this purpose, 20 by default) as well as gene presence/absence profile (pan-genome tree). PanGFR-HM can provide the functional composition (based on COG and KEGG annotations) of core, accessory and unique gene families with over/under representation statistics for genomes of the interest. It is also capable of delivering information about the genes exclusively absent from a specific genome but present in all other genomes within a group, indicating probable gene loss events. Apart from these, another important feature is Pan-CA, which enables users to perform the comparative analyses of pan-genomes and function/pathway annotations of core, accessory and unique genes for up to four user defined groups of pan-genomes.

The web interface for PanGFR-HM has been developed to offer a user-friendly way to access the taxonomic and body-site specific interactive view to explore the divergence in gene repertoire and functional composition among human microbiota. The resource utilizes latest plotting, data storage and computing libraries from various free community resources. All information, including pan-genome profiles, phylogenetic trees (based on both concatenated core genes and gene presence/absence profile), COG and KEGG annotation distribution (for core, accessory and unique gene families), and protein sequences (core, accessory, unique and genes exclusively absent from a particular strain) incorporated in PanGFR-HM are available for download in publication level graphical, tree (newick), table (xls) and text (fasta format of sequences) formats wherever applicable. The protein sequences can be downloaded as representative sets for core/accessory/unique gene families as well as for all the members of each gene family. These sequence files can easily be used further for evolutionary analyses, domain/motif search, study of physicochemical properties etc. PanGFR-HM not only provides novel aspects such as body-site specificity and comparative analysis, but also allows users to choose the genomes of their interest as well as sequence identity criteria for orthology detection. The different levels of sequence identity for orthology prediction allow users to precisely target various evolutionary distances within human microbiota (Pearson, 2013). These features provide PanGFR-HM a ‘dynamic’ status instead of ‘static’ database unlike MetaRef, MicroScope platform and EDGAR 2.0 where, no such user defined options are available. PanGFR-HM is the only dynamic database especially dedicated to human microbiome and integrated huge information with unique functionality compared to its analogs.

### Database Design, Organization and Structure

**FIGURE 1:**
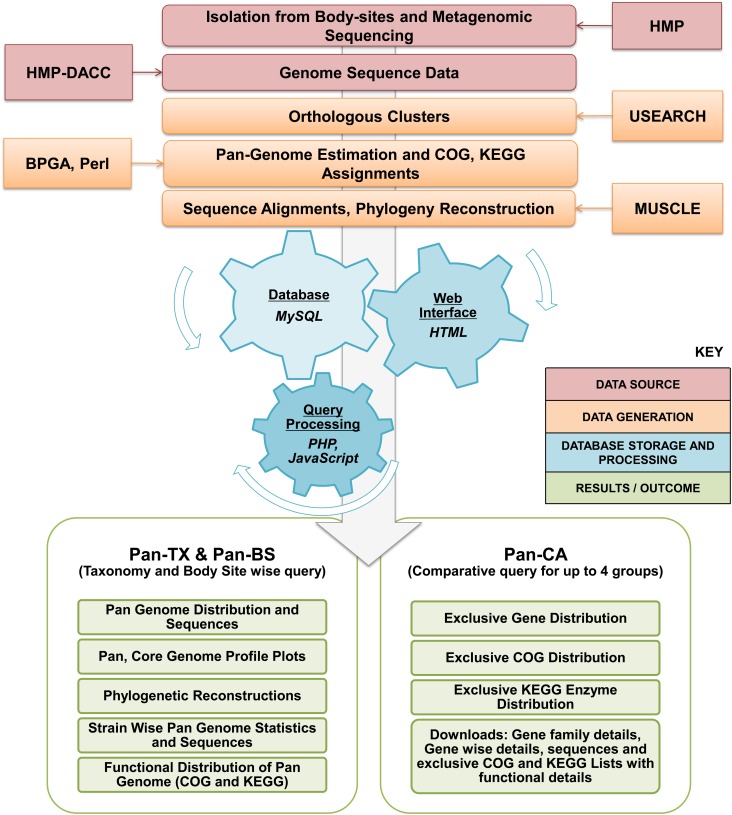
Workflow for PanGFR-HM. The workflow involves preprocessing of the whole genome records from GenBank. The protein sequences from all the available strains are then clustered using USEARCH at various sequence identity cut off levels. The gene presence/absence is determined and stored as a binary matrix using BPGA Pipeline. The tabular data generated after sequence processing and functional assignments are then uploaded into separate MySQL databases for respective sequence identity cut offs. Majority of the backend programs are written in PHP. Finally, various pan genomic and comparative analysis results generated from the set of strains and clustering parameters selected by the users are then provided via the web browser.

The PanGFR-HM logistics has been shown schematically in **Figure 1**. The detailed schema for the database and its connections to the web resource is available in **Supplementary Figure S1**. The resource integrates bacterial and archaeal reference genome data derived from human microbiome and delivers the outcome in the form of pan-genome profile. An easy to use web interface allows users to retrieve the pan-genomic profile and information of functional distribution for any set of available genomes.

For a user defined set of genomes the extrapolation of pan and core genome curves can be performed by empirical power law equations and exponential decay equations respectively as implemented by Bacterial Pan-Genome Analysis Pipeline (BPGA) (Chaudhari et al., 2016). Slope of the power curve (the *B* value), helps users to decide the open/closed nature of pan-genome, i.e., whether the pan-genome size increases considerably after inclusion of additional microbial genome or the saturation is achieved. Phylogenetic analysis can be retrieved from core orthologous clusters and binary presence/absence matrix (pan matrix) using MUSCLE (Edgar, 2004). It first aligns the concatenated protein sequences of core proteins and then builds Neighbor Joining tree upon the alignment. Users can select the number of random core proteins (10, 20, 30, 50, 70, and 100 – default 20) in order to reconstruct the phylogenetic tree. If less number of core proteins than the user-defined core proteins are present, all of them will be considered for phylogenetic tree reconstruction. The overall topology of this random core-genome tree remains unaltered as compared to the tree formed using all core protein sequences when present in large number (Chaudhari et al., 2016). Core, accessory and unique protein families are then assigned for given set of genomes along with their sequences and function/pathway annotations. The functions are annotated using NCBI COG database, 2014 update (Galperin et al., 2015) and KEGG enzymes are annotated using KAAS server (Moriya et al., 2007).

The home page of PanGFR-HM serves as the gateway to the interlinked genomic and functional features. The interface is capable of utilizing the database features dynamically as instructed through interactive web input forms at the respective web modules. The web resource is compatible with the latest versions of Edge (version 41+), Google Chrome (version 66.0+), Safari (version 11.1+), and Mozilla Firefox (version 59.02+).

### Data Generation

The high quality complete genome sequences for 1293 bacteria and archaea were downloaded from HMRGD (HMP Reference Genome sequence Data)^1^. The protein sequences and annotations were extracted from the GenBank records for the same. Protein sequences were clustered separately into orthologous gene families at different sequence identity cut-off values of 40, 50, 60, 70, 80, and 90% using USEARCH (Edgar, 2010). The orthologous clusters were then processed using BPGA (Chaudhari et al., 2016). Using the features of BPGA pipeline, paralogs were discarded for the ease of analysis and binary gene presence/absence matrix was generated. Each orthologous cluster was then mapped with latest NCBI COG database (last updated 2014)^2^ using best blast-hits for annotation of functions and then the assignments of pathways were done by KAAS v2.1 (KEGG Automatic Annotation Server)^3^ using BBH (bi-directional best hit) method using representative protein sequences (Moriya et al., 2007; Galperin et al., 2015).

### Database Creation

All the clustering data along with sequence and function data were integrated into MySQL community database engine (v5.7) in organized manner for each identity cut-off level so that the orthology data can be retrieved based on a query provided by the users.

### Data Processing and Delivery

Web pages were designed in HTML5. User forms and all other calculations including SQL database queries were processed in PHP (v7.0.9) and JavaScript. Most of the plots generated during these analyses used Plotly (v1.29.1), the open source JavaScript graphing library^4^. Sequence alignments and phylogeny trees were generated using MUSCLE (v3.8.31) (Edgar, 2004). Users can also import the phylogenetic trees to iTOL (Interactive Tree Of Life) web server^5^ for better visualizations, formatting and high resolution graphics (Letunic and Bork, 2016). Phylocanvas Library is used for interactive tree visualizations^6^.

### Characterization of Pan-Genome

Pan-genome characterization of group of genomes is a dynamic process and depends upon the criteria for construction of orthologous gene families or clusters generated from clustering tools. We utilized the USEARCH clustering tool (Linux v9.2.64) for all proteins from 1293 currently accessible reference genomes derived from human microbiome at HMRGD^1^. Using PanGFR-HM web form, users can select any number of genomes (maximum 200 genomes recommended) either body-site wise or taxonomy-wise for an analysis, and consider any of the amino acid identity cut-offs (ranging from 40 to 90% with steps of 10) for estimating the orthologous clusters. On the basis of selected identity cut-off value, the respective protein families are then extracted from database along with sequence and functional details to build the pan-genomic and functional profile.

### Functional Over/Under Representation Analysis

For a group of genomes, the differentially represented functional sub categories of each major category of COG and KEGG classification for pan-genome component (core, accessory and unique) proteins are determined based on the respective major category as reference. The statistical analysis for the significance testing is performed using Chi-Square Test with 1 degree of freedom. The following formula is used for calculation of Chi-Square value for a particular sub category within a major category of a specific pan-genome component,

$$x^{2}=\frac{n\cdot {\left(a\cdot b-b\cdot c\right)}^{2}}{\left(a+b)\cdot (c+d)\cdot (a+c)\cdot (b+d\right)}$$

Where, *n* = *a* + *b* + *c* + *d*; a is the count of COG/KEGG assignments of that particular functional sub category and b is the count of the rest of that sub categories of that specific pan-genome component, c and d are the respective counts of COG/KEGG assignments of same functional sub category and rest of the sub categories of remaining two pan-genome components. The functional sub categories which pass the significance test are marked accordingly for over or under representation.

### Methodology for Comparative Analysis

In Pan-CA module the comparative gene analysis is performed in two steps. First, the orthologous gene clusters from all member genomes of each group selected by users are identified and next every possible shared and exclusive gene clusters between the groups are calculated. For example, if users select strains for three groups (A, B, and C) then total seven possible sets will be there: one core set (ABC), three accessory sets (AB, AC, and BC) and three unique sets (A, B, and C). Further the COG/KEGG classification of shared and exclusive gene clusters is presented in both graphical and tabular format. For comparative function analysis and comparative pathway analysis in Pan-CA, only the annotated COG protein identifiers and KEGG enzyme identifiers of all the selected genomes are extracted and pooled instead of gene clusters, followed by group-wise comparison for shared and exclusive COG/KEGG identifiers. All the results are then presented by plotting Venn diagrams (downloadable SVG or PNG images) and providing tabular output with browsing options and downloadable links.

## Results

### Data Overview and Statistics

**FIGURE 2:**
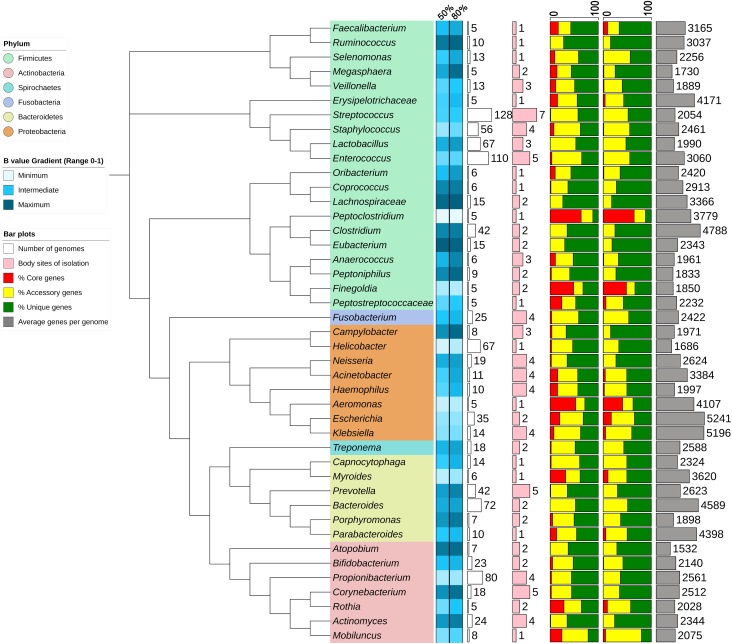
Summary of pan-genome statistics for selected genera. The Neighbor Joining phylogenetic tree is constructed using 16S rRNA genes from representatives of each taxon. The *B* values are depicted as heatmap and the percentages of core, accessory and unique genes as stacked bar plots for each taxon using 50 and 80% sequence identity cut-offs. The plot and heatmap are constructed by iTOL web server using statistics calculated from PanGFR-HM.

The pan-genome statistics of selective genera of human microbiome present in PanGFR-HM are summarized in **Figure 2**. The genera containing at least 5 complete genomes are selected for this analysis. Along with core, accessory and unique gene counts, the figure also depicts the *B* statistic of each pan-genome at both 50 and 80% amino acid sequence identity cut-offs. *B* statistic value gives an idea about the open or closed nature of pan-genome. The *B* value toward ‘1’ indicates the open pan-genome where pan-genome size constantly rises after stepwise addition of new genomes. Whereas, the *B* value toward ‘0’ indicates closed pan-genome where pan-genome size does not change after inclusion of additional genomes.

As shown in **Figure 2**, pan-genomes of the genera *Aeromonas, Finegoldia, Mobiluncus, Myroides, Peptoclostridium*, and *Rothia* seem to add fewer new genes with addition of new genomes with *B* value < 0.4 (Chaudhari et al., 2016). These estimates may be misleading as they are based on predictions from only few available members of a genus (only 5–8 genomes). Whereas, the pan-genomes of genera *Escherichia, Klebsiella, Propionibacterium* and *Staphylococcus* are found to be not growing rapidly with lower *B* values of 0.3/0.33, 0.35/0.38, 0.26/0.31, and 0.39/0.41 based on 35, 14, 80, and 56 genomes at 50/80% sequence identity cut-offs, respectively.

### Query Options

PanGFR-HM offers various features for flexible query and comprehensive pan-genomics as well as comparative analysis of human microbiome strains. The resource can be navigated through any of the three options: (I) Taxonomy-wise Pan-Genome and Functional Analysis, (II) Body-site wise Pan-Genome and Functional Analysis, and (III) Comparative Pan-Genome and Functional Analysis for flexible and rational selection of strains based on various criteria. All of them deliver in-depth analysis of the genomic and functional repertoire of selected strains. Apart from these we have also integrated the BLAST^7^ program within this resource. Therefore users can perform BLAST search for their query sequences against any pan-genomic profile of group of genomes.

The performance of this resource mainly depends on the size of the selected dataset by users and collective server load. The resource took around 15 min for pan-genomic analysis of top 10 genera (based on number of strains present) having total 699 strains run in parallel.

#### Taxonomy-Wise Pan-Genome and Functional Analysis (Pan-TX)

This module enables pan-genomic analysis of any set of the available strains from HMP based on their taxonomy. The users can select all the human microbiome strains from a desired species, genus or any other taxonomic level irrespective of the isolation site within human body. It provides phylogenetic tree reconstruction of the selected strains based on the approaches like pan-genome (gene presence/absence) and core-genome (concatenated and aligned amino acid sequences of core genes) along with the comprehensive pan-genomic and potential functional repertoire of selected taxon.

### Body-Site Wise Pan-Genome and Functional Analysis (Pan-BS)

This module enables users to select microbiome strains for analysis on the basis of their major site of isolation within human body as defined by HMP. Users can also select only the strains isolated from a particular body-site to extract information about gene, function and pathway repertoire among the selected strains along with the routine pan-genomic analysis results.

#### Comparative Pan-Genomic and Functional Analysis (Pan-CA)

The Pan-CA module is another flexible and novel feature of PanGFR-HM. This module enables users to make a flexible query for analysis of up to 4 distinct groups of strains and derive the comparative picture of genes, functions and pathways among selected groups (pan-genomes). The groups can be formed on the basis of taxonomy (like Pan-TX), isolation site of microbes (like Pan-BS) or any other suitable criteria decided by the users.

### Output Options

Pan-genome analysis performed on strains of interest, selected via Pan-TX or Pan-BS, delivers comprehensive pan-genome and functional analyses results. The results include: details of selected strains (dataset), overall pan-genome statistics (proportion of core, accessory, unique genes) for given set of genomes, core and pan-genome profile plots, phylogenetic reconstruction based on core genes and pan-genome, genes specifically absent from individual strain, distribution of proteins in different COG and KEGG functional categories and their over/under representation for each pan-genomic component, and strain wise pan-genome statistics along with data or sequence download links for all plots, phylogenetic trees and protein sequences etc.

The comparative analyses performed in Pan-CA module on groups of microbiome strains of interest provide results for orthologous proteins, COG identifiers and KEGG enzyme identifiers for all possible sets (shared and unique) between up to four groups. Distribution of proteins or identifiers in every possible set is explained with Venn diagrams, and data for each of these sets is provided as spreadsheets. For comparative analysis of orthologous proteins downloadable FASTA sequences for further analyses and COG/KEGG classification details with plots are also given.

When BLAST search is performed with protein sequences uploaded by users, it generates mainly two kinds of outputs. One of them includes pan-genomic distribution plot of gene clusters from selected strains for building the database. The other depicts the BLAST output spreadsheet showing how many proteins among the queried proteins have pan-genomic orthologs along with pan-genomic status (core/accessory/unique), KEGG identifiers, COG identifiers, sequence alignment details etc. For each orthologous proteins clickable links are given to corresponding alignments, COG/KEGG and gene identifiers details. Also a distribution plot is available summarizing pan-genomic distribution of orthologous proteins.

### Additional Novel Features

#### Dynamic Estimation of Pan-Genome

Pan-genome characterization of a group of genomes is a dynamic process, which greatly depends upon the criteria for construction of orthologous gene families or clusters generated from the sequence clustering tools. The pan-genome estimation may highly fluctuate for different sequence identity cut-off criteria depending upon the rate of divergence, although overall pan-genome characteristic does not vary much for closely related genomes (Paul et al., 2016). Using PanGFR-HM web form, users may select at least 5 to all genomes (maximum 200 recommended, for more than that the resource will take longer time) at a time, and proceed for analysis based on various sequence identity cut-offs ranging from 40 to 90% for constructing orthologous protein clusters. This feature brands PanGFR-HM as a dynamic server, not just a static database with pre-calculated clusters with fixed parameters.

#### Exclusive Absence of Genes: A Clue to Gene Loss Events

It is well known that bacterial genomes acquire new genes from surrounding gene pools to get an adaptive advantage to the environmental or cellular changes (Dutta and Pan, 2002; Popa et al., 2011; Arber, 2014; Li et al., 2014). Most of these genes fall under unique genes category in any pan-genome analysis due to lack of orthologs in related organisms. Apart from these unique genes, another very important evolutionary process is gene loss, which may be another adaptive strategy for genome evolution (Hottes et al., 2013; Bolotin and Hershberg, 2015). The gene loss events are often hard to track down at sequence level. A novel feature is integrated in PanGFR-HM for investigating the genes exclusively absent (not matching under given sequence identity cut off) from a genome but present in all other genomes of the users selected dataset. By exclusive gene absence analysis in PanGFR-HM, one can estimate such probable events, *in silico*. These exclusively absent genes might also be important for adaptation of the microbes at a specific niche. PanGFR-HM specifically extracts those gene families and provides their sequences for download and function annotations.

#### Functional Over/Under Representation Analysis

The assignments of COG and KEGG functional classification are done for core, accessory and unique gene sets. The significantly over and underrepresented functional categories within a major category among the above sets are reported. The feature aids in understanding the gene divergences which led to the functional evolution of pan-genome.

#### BLAST Search Against Pan-Genomic Profile

This feature allows users to paste/upload their own protein sequences in FASTA format and perform the BLAST search against user defined pan-genomic profile from PanGFR-HM. Users have the option to select strains of interest (either based on taxonomy or isolation site) in order to create a representative set of pan-genomic profile, which will be used as database for BLAST search. Therefore, if the query sequences have orthologous proteins in pan-genome set, the queried proteins will be annotated accordingly. Thus, by performing the BLAST search against any user-defined pan-genomic profile for all the proteins in any new genome of interest, it is possible to define the core, accessory and unique proteins of that new genome.

### Demonstration of Comparative Analysis and Its Applications

For demonstration of Pan-CA Module, we considered all available *Lactobacillus* strains from human microbiome and divided them into three groups according to their major body-site of isolation, i.e., human gastrointestinal tracts (gut), oral cavity and urogenital tracts. The summary of selected dataset is shown in **Table 1**. The complete list of strains used for this analysis is provided in **Supplementary Table S2**.

**Table 1:** Summary dataset of *Lactobacillus* strains used for comparative analysis.

Body site	No. of strains	Total proteins	Average proteins per genome
Gastrointestinal tract	23	53687	2334
Oral cavity	4	10534	2634
Urogenital tract	40	69081	1727


These three groups are provided as input for comparative analysis to retrieve group specific exclusive sets of gene families, KEGG enzymes, and COG annotated proteins. The analysis reveals interesting trend about the peculiar gene/function repertoire of these three groups and created a comparative evolutionary portrait of *Lactobacillus* strains at the distinct body-sites.

#### The Gene Family Distribution

**FIGURE 3:**
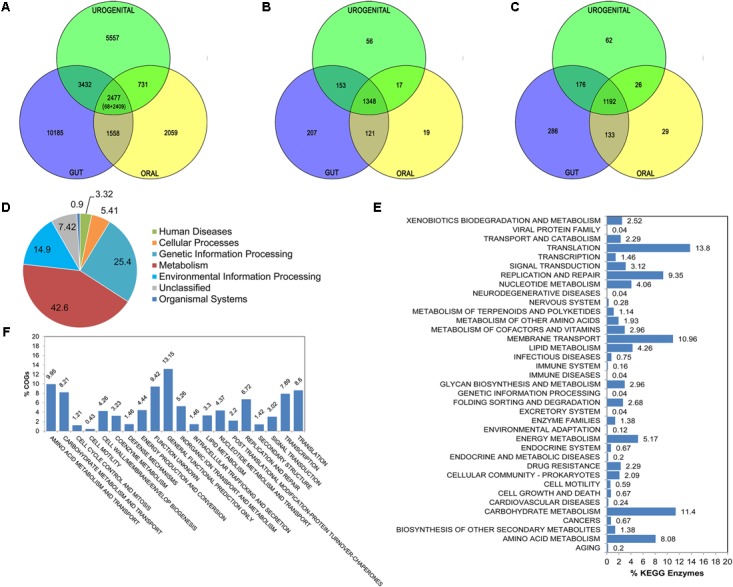
Comparative analysis for *Lactobacillus* strains from gut, oral cavity and urogenital tracts. Venn diagrams depict the number of gene families **(A)**, COG proteins **(B)** and KEGG enzymes **(C)** at three body-sites. Distribution of major KEGG functional categories for 1192 core enzymes is shown as pie chart **(D)**. Bar plots are created in order to illustrate the distribution of detailed core functional KEGG enzymes **(E)** and COG proteins **(F)**.

The complete set of proteins upon clustering (using sequence identity cut-off of 50%) generates the protein families for all members of three groups. The group specific exclusive sets are calculated along with all other possible combinations between groups. Then the shared and exclusive gene sets are extracted with sequences. As shown in **Figure 3A**, there are 2477 gene families which contain proteins from at least one member from each body-site. Out of these 2477 gene families, 68 gene families are found in all the 67 *Lactobacillus* strains irrespective of body-sites representing the *absolute core*; most of them are involved in house-keeping functions like *translation* and *cell wall/membrane/envelop biogenesis*. While, the remaining 2409 gene families represent *extended core* set. There are 10185, 5557 and 2059 gene families specific for gut, urogenital tract and oral cavity respectively (**Figure 3A**).

#### The COG Function Distribution

The comparison of COG identifiers pooled together for each *Lactobacillus* group provides exclusive COG functions present at respective body-site at annotation level; irrespective of strain details. **Figure 3B** shows the distribution of COGs between the three *Lactobacillus* sets. There are total 1348 COGs common to all the three *Lactobacillus* groups, i.e., core in nature. As shown in **Figure 3F**, most of the Core COGs fall under *Amino acid transport and metabolism, Translation* and *Carbohydrate transport and metabolism*. The distributions of body-site specific functional categories are also retrieved through Pan-CA module (see **Supplementary Figures S2–S4**).

#### The KEGG Enzyme Distribution

The comparison of KEGG enzymes pooled together for each *Lactobacillus* group provides exclusive pathway profile present at respective body-site at pathway level; irrespective of strain details (**Figure 3C**). Most out of these 1192 core enzymes are involved in *Metabolism* and *Genetic information processing* (**Figure 3D**). Upon detailed analysis, the proportions of genes in *Translation, Carbohydrate metabolism*, and *Membrane transport* pathway categories are found to be high within these core enzymes (**Figure 3E**).

The results also reveal about gut, oral cavity and urogenital tract specific enzymes among *Lactobacillus* strains (see **Supplementary Figures S5–S10**). Overall, the gut, oral and urogenital tract specific enzymes show highest proportion of *Membrane transport* related pathways. However, the *Cell motility* pathways are highly represented in gut specific Lactobacilli; this is in conformation of previous reports suggesting biological significance for presence of cell motility in gut bacteria which may potentially favor better acquisition of nutrients and successful colonization to the niche environment (Cousin et al., 2015). *Cancer* related pathways are present in oral Lactobacilli only, indicating possible role of oral microbiota in carcinogenesis (Meurman, 2010). *Signal transduction* related pathways are in higher proportion in urogenital tract Lactobacilli as compared to those from other body-sites, also reported previously (Mendes-Soares et al., 2014). Such body-site specific enzyme sets might be involved in body-site specific adaptive strategies during human-microbe co-evolution.

### Comparison of PanGFR-HM With Other Resources

PanGFR-HM is the only resource providing comprehensive pan-genomic analysis exclusively for the human microbiome strains. Also, as per our knowledge, no resource provides online comparative gene, COG/KEGG classification analysis of user-defined groups of microbiome strains. However, some related resources are considered here for overall comparison of pan-genomic output on microbial data irrespective of their relation to human microbiome context. The details can be accessed from **Table 2**.

**Table 2:** Comparison of PanGFR-HM with other microbial pan-genome analysis resources.

Functional feature	PanGFR-HM^†^	EDGAR 2.0	Micro Scope	MetaRef
No. of genomes included	1293 (S.)	2160 (N.S.)	3871 (N.S.)	2818 (N.S.)
Pan-genome distribution	✓	✓	✓	✓
Pan, core profile (development) plots	✓	✓	✓	×
Strain wise pan-genome distribution	✓	✓	✓	×
Core-genome based phylogeny	✓	✓	×	×
Forming groups of strains	✓	✓	×	×
Sequence identity cut-off setting option	✓^#^	×	✓^∗^	×
*Editable and interactive plots*	✓	×	×	×
*Pan-genome based phylogeny*	✓	×	×	×
*Strain wise sequence retrieval from pan-genome*	✓	×	×	×
*Exclusive gene distribution*	✓	×	×	×
*Exclusive COG and KEGG distribution*	✓	×	×	×
*Over/under-representation of functional classes*	✓	×	×	×
*Exclusive absence of genes*	✓	×	×	×
*COG and KEGG distribution of pan-genome*	✓	×	×	×


*^†^Present tool, ^#^40–90% (40, 50, 60, 70, 80, and 90%) identity cut-off options available, ^∗^Only 50 and 80% identity cut-off option available, S., Specific to human microbiome, N.S., Non Specific, ✓-present, ×-absent. Features listed in italics are exclusive to PanGFR-HM.*

## Discussion

The prime objective of PanGFR-HM was to create a user friendly dynamic platform, which applies concept of pan-genome to better understand genomic/functional repertoire of inhabitant microbes of the human microbiome. This web resource is equipped with unique features to extrapolate the genomic data to speed up and simplify pan-genomic and functional comparative analyses on large datasets of reference microbes from the human body.

### Limitations of the Pan-Genome Construction Methods

In cases of orthology based pan-genome approaches, the sequence identity cut-off is the critical parameter which determines if the given gene family belongs to conserved genome or dispensable genome. Larger changes in the cut-off values may considerably change the status of gene family. The higher identity cut-off (more than 70%) may reduce the ‘core’ set and increase the accessory or strain specific gene sets. On the other hand, lower identity cut-off used for exactly same dataset will allow more genes to be assigned as core genes based on lower threshold for ortholog prediction. Also, the protein diversity within a selected taxon, clade or dataset is one of the factors for deciding appropriate identity cut-off. The members of same species are closely related in taxonomic and evolutionary aspects. They need higher identity cut-offs to establish the orthology in order to reveal recent evolutionary changes. As we move from specific taxonomic levels like species to genus or more general ones, the members become distant in terms of genome evolution, so, lower identity cut offs are recommended. So, the default 50% used for PanGFR-HM seems optimal for related organisms up to genus or family level, but again the genome diversity characteristics of each genus or family may vary. The users need to set these parameters with caution.

### Availability of Complete Genomes for Human Microbiota

The present dataset of completely sequenced microbial genomes isolated from human body specific sources may not represent the complete picture of the microbiome, it will always remain a work in progress for a while. The advantage of pan-genome based concept is that it hints you toward the sequencing effort needed for certain taxa, i.e., whether the number of strains used in pan-genome are sufficient to explain the genomic architecture of particular taxon. For taxa showing open pan-genomes need more and more completed genomes of its members for more comprehensive genomic landscape of those taxa, while the near-closed pan-genome suggests limited gene acquisition and loss within that taxon.

## Conclusion

This resource will encourage researchers to study essential and ubiquitous microbiota at various taxonomic levels and enable them to gaze into the intricate functional and pathway details of specific groups of microbiome communities. Currently, the resource is focused to the genomic/functional repertoire of completely sequenced microbial genomes from HMP, and in future we plan to make the database more resourceful with each update by incorporating new complete genomes, draft genomes and genomes from other sources. As there will be additional newly sequenced complete microbiome stains available through human microbiome or other microbiome projects we plan to update the database contents twice a year to accommodate those strains. Obviously, the more the reference genomes better will be the overall representation of pan-genomic features. PanGFR-HM is committed to accommodate the expanding taxonomic and genomic landscape of the human microbiome.

## Availability of Supporting Data and Materials

The resource can be freely accessed at http://www.bioinfo.iicb.res.in/pangfr-hm/. All the complete genomes used for generation of PanGFR-HM were publically available from https://www.hmpdacc.org/HMRGD/ (The complete list of genomes used for PanGFR-HM is available in **Supplementary Table S1**). The case study results on 67 *Lactobacillus* strains can be reproduced from http://www.bioinfo.iicb.res.in/pangfr-hm/pan-ca.html, by selecting the strains listed in **Supplementary Table S2**.

## Author Contributions

NC and VG conceptualized the project and drafted manuscript. NC, VG, AG, and GK generated pan genome database from raw data. NC and AG did the required programming. CD added thoughtful suggestions during the work and manuscript writing. SP conceived and coordinated the project, and revised the manuscript. All the authors read and approved the final manuscript.

## Conflict of Interest Statement

The authors declare that the research was conducted in the absence of any commercial or financial relationships that could be construed as a potential conflict of interest.

## Footnotes

1 https://www.hmpdacc.org/HMRGD/

2 ftp://ftp.ncbi.nih.gov/pub/COG/COG2014/data/

3 http://www.genome.jp/tools/kaas/

4 https://plot.ly/javascript/

5 https://itol.embl.de/

6 http://phylocanvas.org/

7 https://blast.ncbi.nlm.nih.gov/Blast.cgi
