# Supplementary material for: PanGFR-HM: A Dynamic Web Resource for Pan-Genomic and Functional Profiling of Human Microbiome With Comparative Features
Source: Front Microbiol. 2018 Oct 8;9:2322. doi: 10.3389/fmicb.2018.02322 (PMC6187978; doi:10.3389/fmicb.2018.02322)
Supplement: Supplementary file 1 [file Data_Sheet_1.PDF]

Supplementary File 2

PanGFR-HM: a dynamic web resource for pan-genomic and functional profiling of human microbiome with comparative features.

Narendrakumar M. Chaudhari, Anupam Gautam, Vinod Kumar Gupta, Gagneet Kaur, Chitra Dutta and Sandip Paul*

*** Correspondence:** sandippaul@iicb.res.in and websandip@gmail.com

All the plots in this file were generated through Pan-CA module of PanGFR-HM.

Supplementary Figures

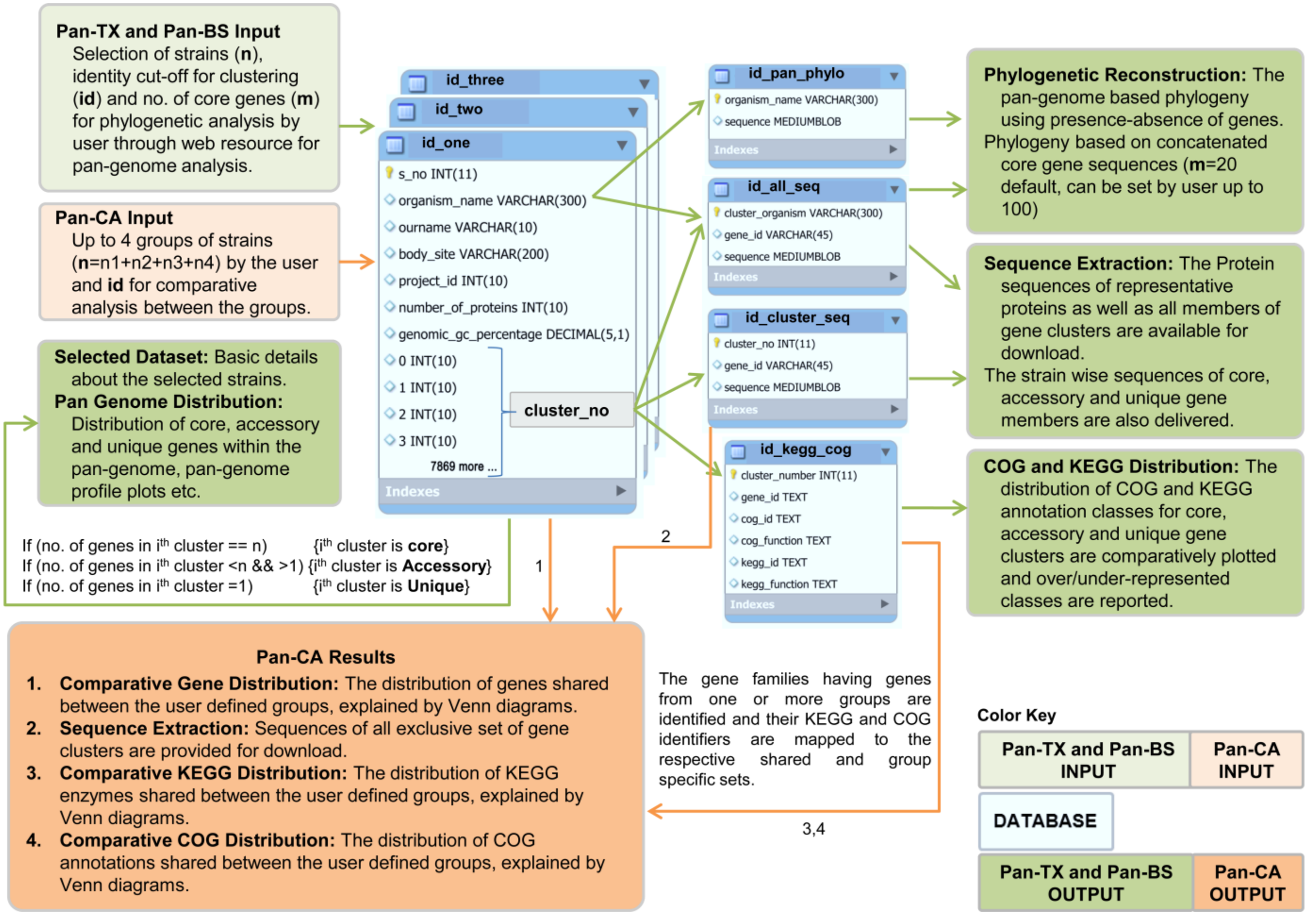

Figure S1: Database Schema.

The database schema design for pan genomic data generated by 1293 strains at 50% sequence identity cut-off is shown. ‘n’ stands for the total number of genomes selected by the user for pan-genomic or comparative analysis. The exactly same schema works for each of the other identity cut-off (id) values i.e. 40, 60, 70, 80, and 90%. The selected identity cut-off by user will initiate the query into respective schema.

In short, the selected organisms are queried into the respective cluster database table (id_one, id_two, id_three) to get the list of clusters from the selected genomes. The gene presence absence table (id_pan_phylo) are used for Pan Genome Phylogeny. The core, accessory and unique gene families are identified and queried into the KEGG and COG table (id_kegg_cog) to obtain the distribution of COG and KEGG categories within the pan genome. The sequence tables (id_all_seq, id_cluster_seq) to get representative as well as all sequences from the these clusters. The core protein sequences from each genome are utilizes for Core Genome Phylogeny.

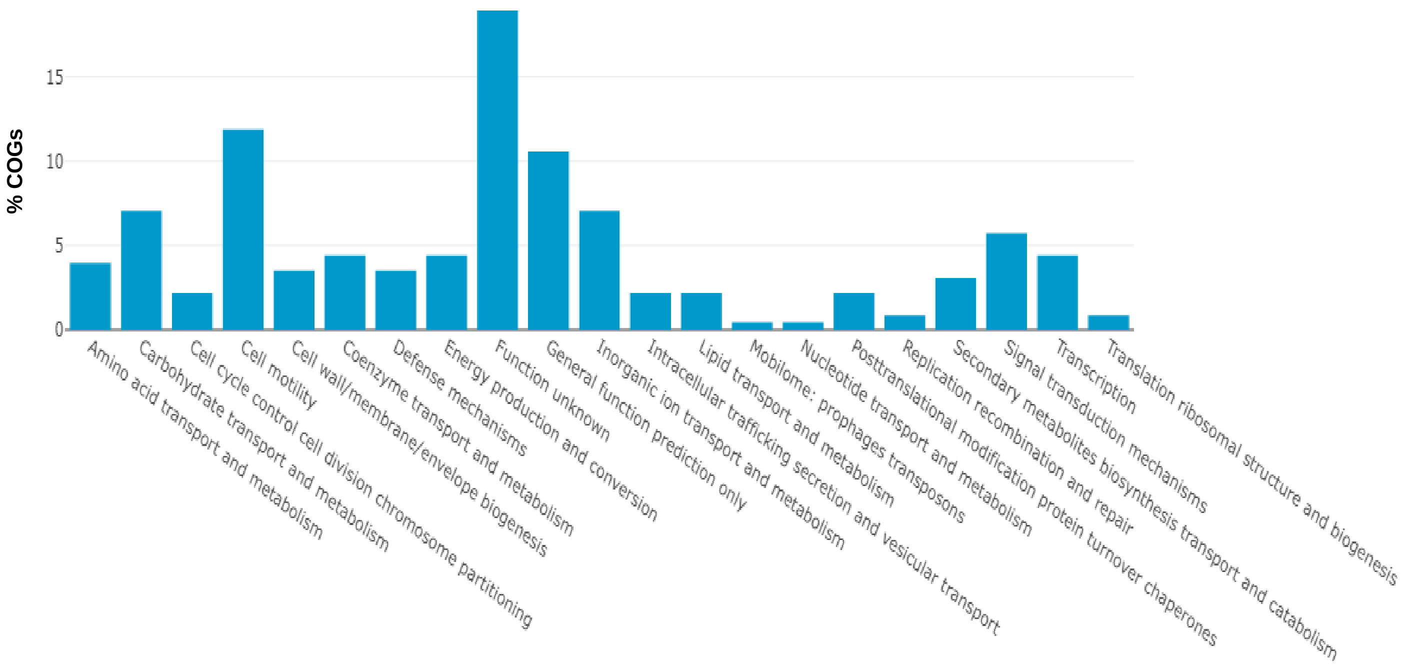

Figure S2: COG distribution for specific genes in Lactobacillus strains from gut.

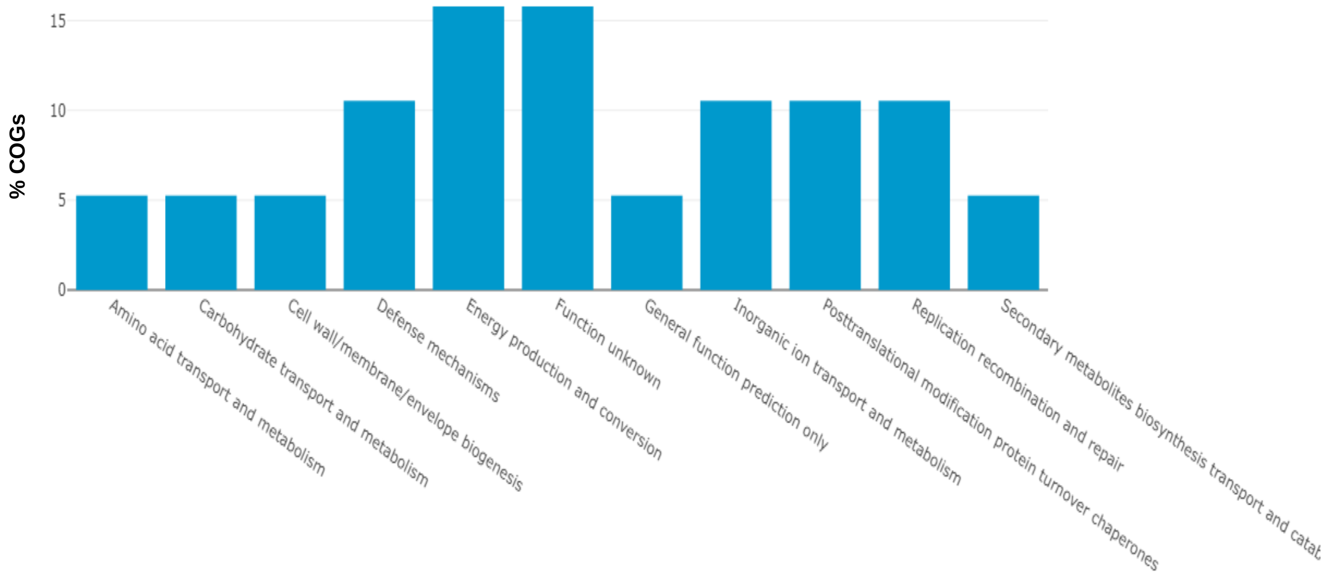

Figure S3: COG distribution for specific genes in Lactobacillus strains from oral cavity.

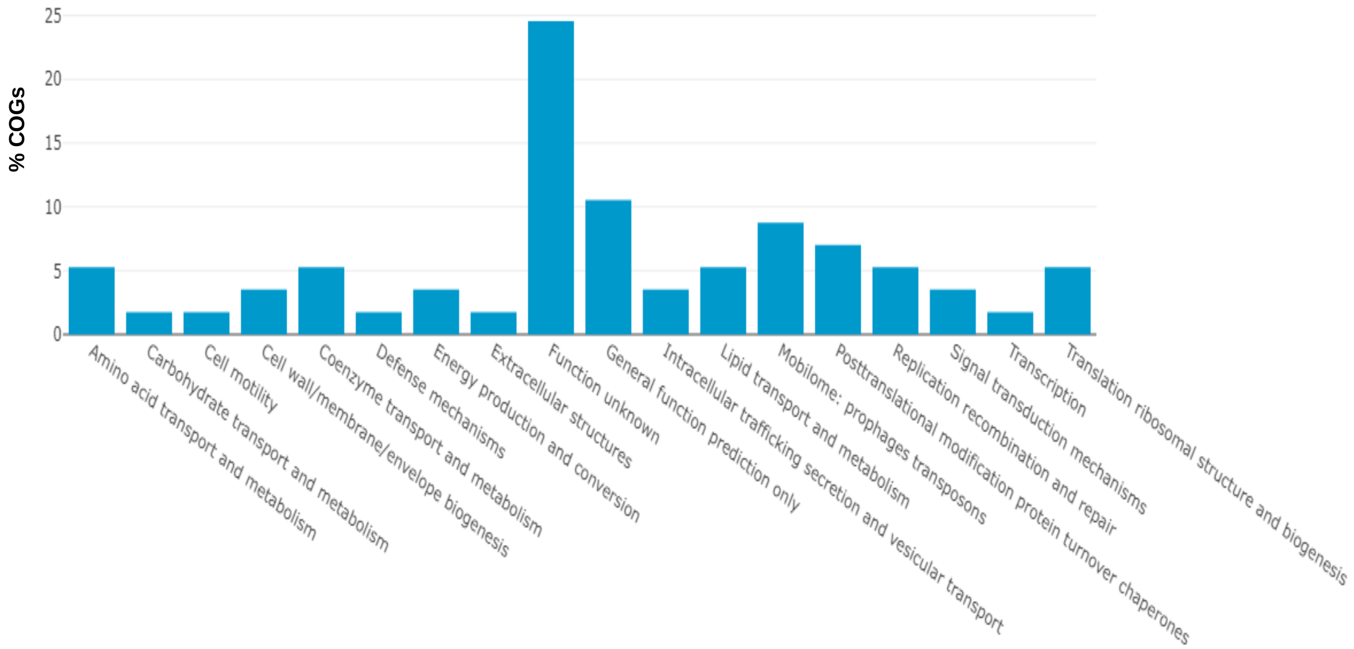

Figure S4: COG distribution for specific genes in Lactobacillus strains from urogenital tracts.

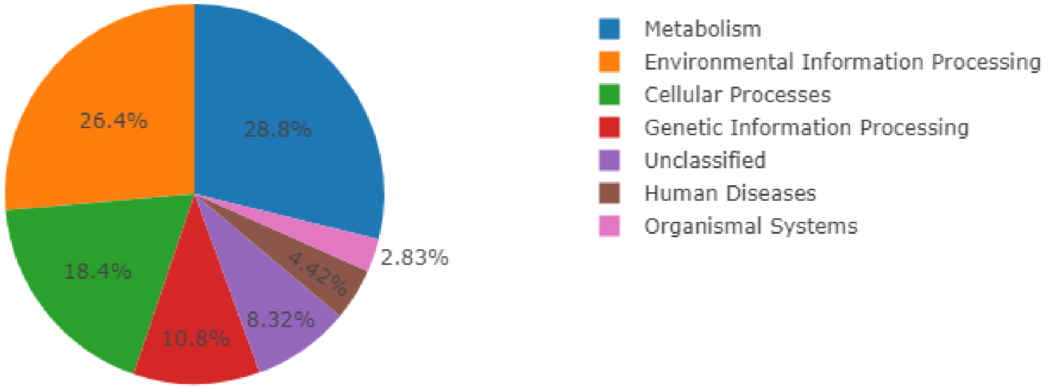

Figure S5: KEGG distribution for specific genes in Lactobacillus strains from gut.

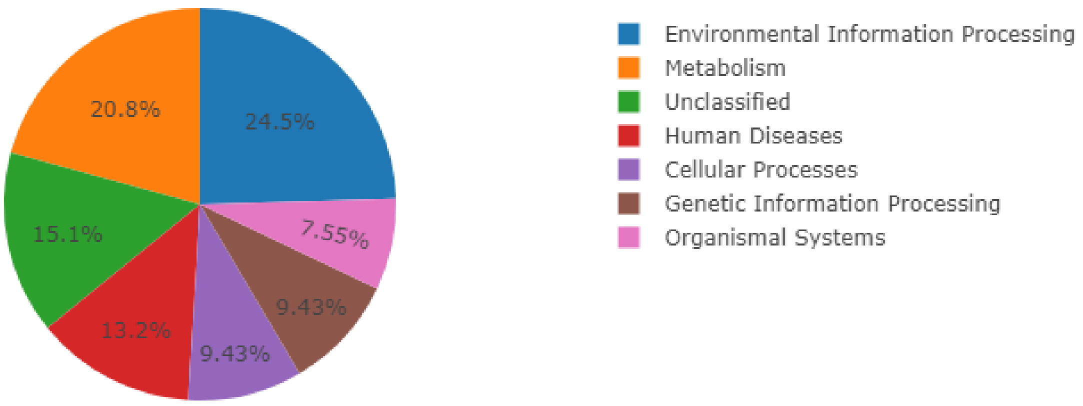

Figure S6: KEGG distribution for specific genes in Lactobacillus strains from oral cavity.

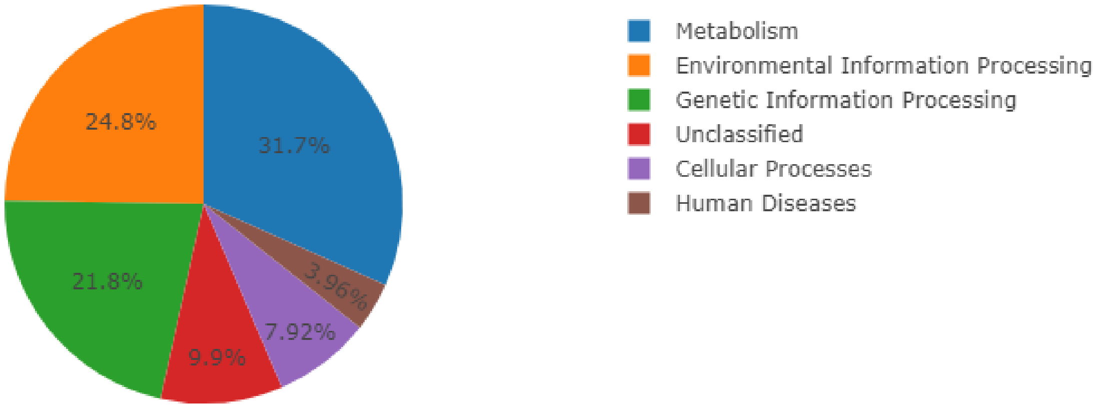

Figure S7: KEGG distribution for specific genes in Lactobacillus strains from urogenital tracts.

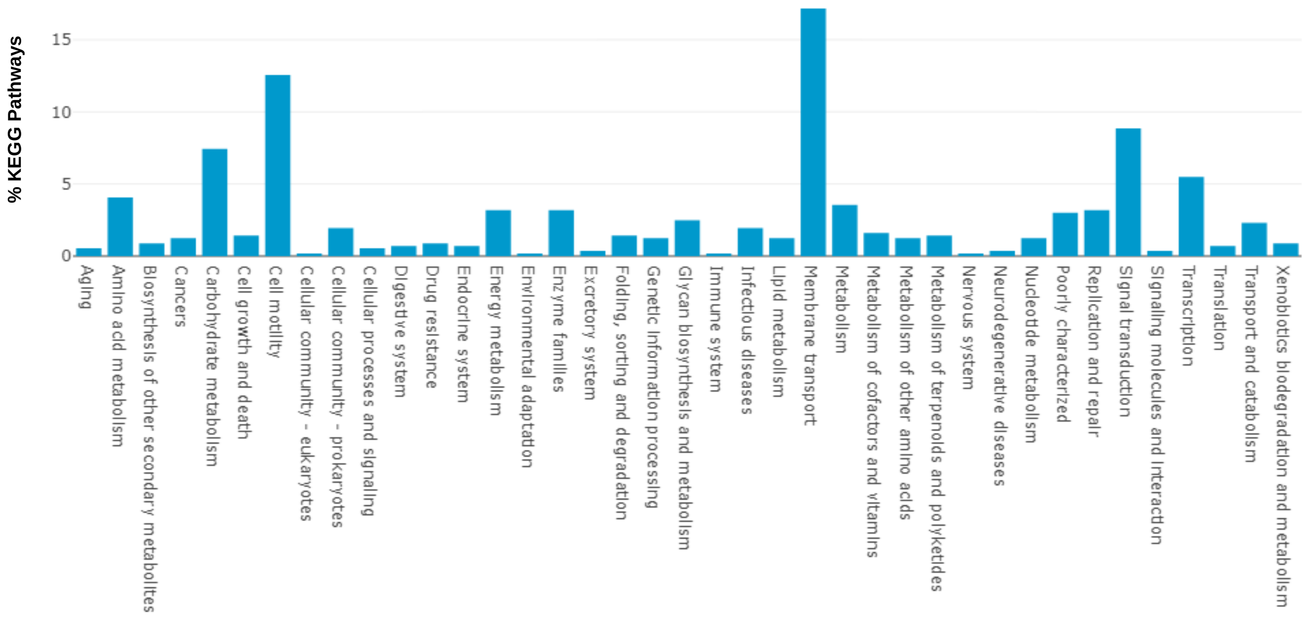

Figure S8: Detail KEGG distribution for specific genes in Lactobacillus strains from gut.

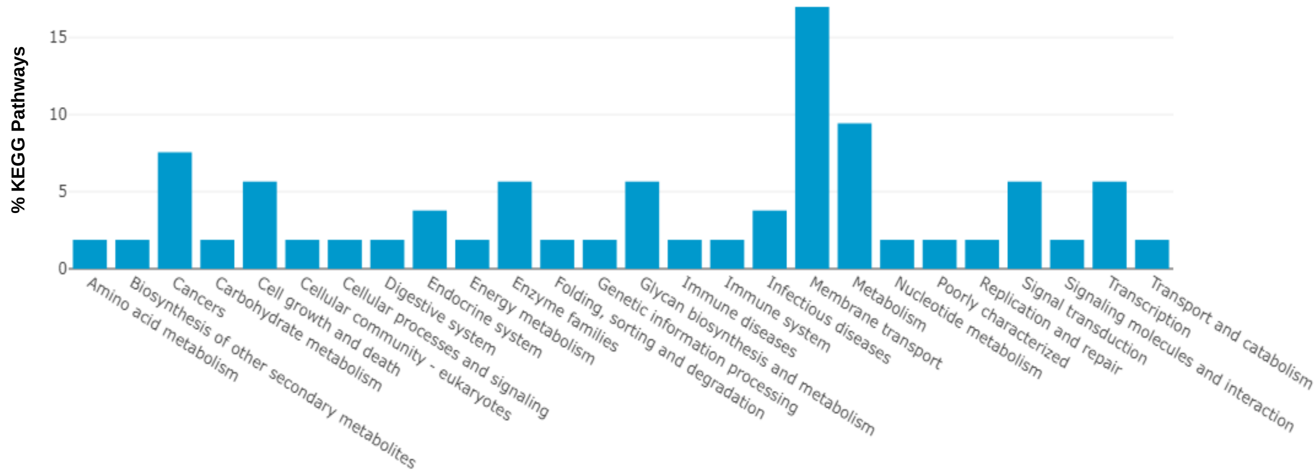

Figure S9: Detail KEGG distribution for specific genes in Lactobacillus strains from oral cavity.

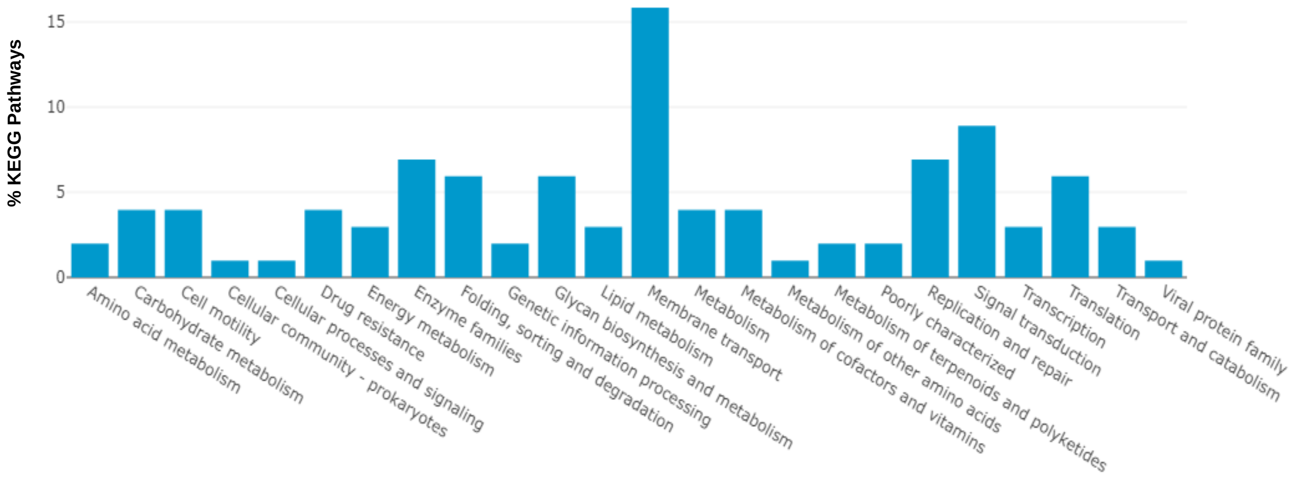

Figure S10: Detail KEGG distribution for specific genes in Lactobacillus strains from urogenital tracts.
